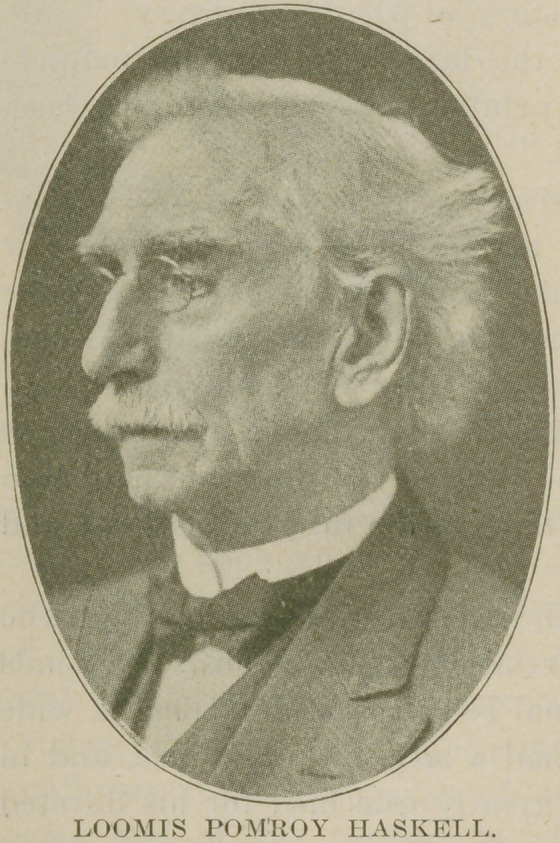# Dr. Loomis Pomroy Haskell

**Published:** 1916-11

**Authors:** 


Dr. Loomis Pomroy Haskell was born April 25, 1826, in Bangor, Maine. His early education was secured in the public schools of Salem, Mass., where his parents moved when he was a child. As a boy he began work in a printing office and a few years ago was banqueted as the oldest living printer. In 1845 he began the practice of dentistry in Boston, making prosthetic dentistry a specialty. In 1856 he moved to Milwaukee, Wis., and one year later came to Chicago, where he has continued in practice ever since. Dr. Haskell for a number of years was the Professor of Prosthetic Dentistry in the Chicago College of Dental Surgery, and afterwards held similar position in Northwestern Dental College.

LOOMIS POMROY HASKELL.

He founded and conducted the Haskell Postgraduate School of Prosthetic Dentistry for ten years.

In 1898 and 1899 Dr. Haskell conducted postgraduate classes in Berlin and Hamburg, Germany; Vienna, Austria, and Paris, France.

In 1863 and 1864 Dr. Haskell published the *People's Dental Journal—Quarterly* and for over forty years has been a liberal contributor to nearly all of the dental journals. His intimate association with the pioneers of the dental world furnished a fund of interesting anecdotes, which he delighted to relate to beginners, with the purpose of making them appreciate the great advantages of the present age, educationally and otherwise, which they have the opportunity to enjoy.

Dr. Haskell was one of the founders of the Illinois State Dental Society in 1864, and was the recipient of many honors. On the occasion of his ninetieth birthday he was the honored guest at a banquet, and with the exception of the loss of the sight of one eye, due to cataract he looked the picture of health. His death was due to pneumonia, which developed after undergoing two surgical operations. The funeral service was conducted in the Congregational Church, at Hinsdale, Ill., of which he was an active member, and was attended by twenty-four members of the Chicago Dental Society, who officiated as active and honorary pallbearers, and many friends—G. N. West, in *Dental Review.*

## Figures and Tables

**Figure f1:**